# Is Barthel Index Suitable for Assessing Activities of Daily Living in Patients With Dementia?

**DOI:** 10.3389/fpsyt.2020.00282

**Published:** 2020-05-08

**Authors:** Yayan Yi, Lin Ding, Huangliang Wen, Jialan Wu, Kiyoko Makimoto, Xiaoyan Liao

**Affiliations:** ^1^Department of Nursing, Zengcheng Branch, Nanfang Hospital, Southern Medical University, Guangzhou, China; ^2^Department of Nursing, School of Nursing and Rehabilitation, Konan Women’s University, Kobe, Japan

**Keywords:** activities of daily living, Barthel Index, dementia, long-term care, Rasch analysis

## Abstract

**Objectives:**

To evaluate application of the Barthel Index (BI) in assessing basic activities of daily living (ADL) of patients with dementia using Rasch analysis.

**Design:**

A multi-country cross-sectional study.

**Setting and Participants:**

Nineteen long-term care facilities located in China, Japan, South Korea, and Thailand. A total of 644 patients with dementia were included.

**Methods:**

Unidimensionality, global and item fit, local dependence, person-item targeting, threshold disordering, and differential item functioning (DIF) were examined. Negative correlations between scores for DIF items and Neuropsychiatric Inventory Nursing Home version (NPI-NH) were evaluated.

**Results:**

Item reliability (1.0) and person reliability (.88) were acceptable. The Rasch dimension explained 72.9% of the variance (Eigenvalue = 27), while the first contrast explained 6.6% (Eigenvalue = 2.4). The “mobility” was misfitting to the Rasch model (infit mean square = 1.86). The overall difficulty of the BI exceeded patients’ ability (person location = −2.27 logits). The “stairs climbing” and “mobility” showed narrow category thresholds (< 1.4 logits). The location of “controlling bladder” and “toilet use” overlapped. Removing “stairs climbing”, collapsing categories with narrow threshold widths in “mobility”, and combining “controlling bowel” and “controlling bladder” into one item, improved unidimensionality, and item fit of the scale. Only three items (“grooming”, “dressing”, and “toilet use”) were free from DIF across countries. The scores for “feeding” were negatively related to scores for “disinhibition” (r = −0.46, P < 0.01), and scores for “controlling bowel” were negatively related to scores for “disinhibition” (r = −0.44, P < 0.01), “agitation” (r = −0.32, P < 0.05), and “aggression” (r = −0.27, P < 0.01) in Japanese samples.

**Conclusions and Implications:**

The performance of the BI for assessing patients with dementia might be compromised by misfit items, person–item mistargeting, measurement gaps, redundant items, narrow threshold width, and item bias. Mobility ability might not be helpful for determining capability of basic ADL in the patients. Comparisons of BI scores between countries should be undertaken with caution due to item bias. Neuropsychiatric symptoms might interact with basic ADL abilities of the patients. We will not suggest using the instrument in patients with dementia, without future refining to improve its performance.

## Introduction

Progressive decline in the ability to perform activities of daily living (ADL) and subsequent loss of independence are the defining features and most severe characteristics of dementia (1–3). Assessment of ADL is important when measuring disease severity (4), evaluating interventions, and determining care needs (5) in patients with dementia. However, there is currently no general consensus regarding instruments for ADL evaluation in patients with dementia (1, 6). Although some instruments have been specifically designed to assess ADL in patients with dementia (7–9), time-consuming and incomplete validation restrict their use in clinical practice or epidemiological studies (1, 10).

The Barthel Index (BI) is a widely used measure of basic ADL function (self-maintenance skills such as dressing, bathing, and grooming), because of its simplicity, communicability, and ease of scoring (11, 12). Similar to other ADL instruments, the items of the BI possess a hierarchy of difficulty and yield ordinal intervals between adjacent scores (13). Therefore, practitioners and researchers may have difficulty interpreting the clinical meaning of BI summary scores or changes in scores. Although the BI has been validated and is used globally, empirical evidence for its validity for assessing patients with dementia is scarce.

Rasch analysis, a probabilistic mathematical modeling technique based on item response theory, is based on two assumptions: 1) that an instrument should be unidimensional such that items measure a single underlying construct; and 2) that scores on a measure depend on both person ability and item difficulty. If data fit a Rasch model, for the same person ability, the probability of endorsing an easy item must be higher than the probability of endorsing a more difficult item and *vice versa* (14, 15). Rasch analysis can transform ordinal data into equal interval measures expressed in linear log-odds probability units and therefore may provide interpretable information of the instrument in evaluating a targeting population. Rasch analysis focuses on individual items in an instrument rather than summary scores and therefore may provide diagnostic techniques for identifying ways of improving items. To the best of our knowledge, Rasch analysis of BI data from patients with dementia has not yet been performed.

The present study investigated the application of the BI for assessing basic ADL in patients with dementia using Rasch analysis, aiming to provide diagnostic information for further revision of the instrument.

## Methods

### Study Design

This cross-sectional study was part of the Prevalence of SymptOms of DementIa in East-Asian Cross-cultural (ePiSODIC) study comparing the type and prevalence of neuropsychiatric symptoms in patients with dementia residing in long-term care facilities (LTCFs) in East Asia. A detailed description of The ePiSODIC has been presented elsewhere (16).

### Setting

Data were collected between September 2015 and April 2016 from study sites located in four countries and one district (**Table 1**). Among the study sites, 19 facilities were recruited, including one dementia care unit in a nursing home in southern China, nine group homes in a major metropolitan area of Japan, two special dementia care units in a general hospital in Western Japan, two LTCFs in Central Japan, one nursing home and one group home in a metropolitan area in South Korea, one nursing home in Taiwan district, and three nursing homes in Thailand.

**Table 1:** Demographic and clinical characteristics of the participants.

Variables	Total (n = 644)	M-China (n = 154)	Japan (n = 235)	S-Korea (n = 97)	Taiwan (n = 97)	Thailand (n = 61)
Female, n (%)	375 (58.2)	99 (64.3)	170 (72.3)	75 (77.3)	0 (0)	31 (50.8)
Age (yr), mean ± SD	83.42 ± 7.67	81.25 ± 7.73	84.30 ± 7.31	84.75 ± 7.15	87.23 ± 6.23	77.38 ± 6.92
Education (yr), mean ± SD	3.36 ± 1.61	2.86 ± 1.38	4.50 ± 1.45	3.01 ± 1.46	2.58 ± 0.73	1.82 ± 0.93
MMSE, mean ± SD	10.10 ± 7.31	7.11 ± 7.23	11.14 ± 7.59	8.81 ± 6.96	13.98 ± 6.09	9.95 ± 5.14
CDR = 1, n (%)	157 (24.4)	19 (12.3)	65 (27.7)	7 (7.2)	42 (43.3)	24 (39.3)
CDR = 2, n (%)	226 (35.1)	54 (35.1)	85 (36.2)	32 (33.0)	35 (36.1)	20 (32.8)
CDR = 3, n (%)	261 (40.5)	81 (52.6)	85 (36.2)	58 (59.8)	20 (20.6)	17 (27.9)
BI score, mean ± SD	9.55 ± 6.27	8.21 ± 6.41	9.23 ± 5.83	5.28 ± 5.11	12.62 ± 3.68	16.04 ± 5.22
Length of stay (month), mean ± SD	39.37 ± 40.79	44.51 ± 36.93	39.67 ± 43.91	29.36 ± 37.25	31.61 ± 23.73	54.13 ± 9.52
NPI-NH score, mean ± SD	15.16 ± 13.19	16.67 ± 12.71	15.03 ± 13.08	18.84 ± 15.43	10.83 ± 13.09	11.29 ± 9.01

M-China, mainland China; S-Korea, South Korea; MMSE, Mini-Mental State Exam; CDR, Clinical Dementia rating; BI, Barthel Index; NPI-NH, Neuropsychiatric inventory-nursing home. Missing data: MMSE (n = 26, 4.0%) and NPI-NH (n = 138, 21.4%).

### Participants

The eligibility criteria of participants were as follows: 1) age ≥ 60 years; 2) admitted to a facility dedicated to caring for patients with dementia; 3) diagnosed with dementia or with a Clinical Dementia Rating (CDR) score ≥ 1; 4) on a stable dose of psychotropic medication and clinically stable for at least 2 weeks prior to the study. Exclusion criteria were as follows: 1) past or present comorbidity of any other psychotic disorder; 2) complication with another end-stage disease; 3) the patient or their legal guardian refused to participate in this study.

The diagnostic criteria for dementia were based on the International Classification of Diseases, 10th Revision, Diagnostic and Statistical Manual of Mental Disorders, 4th edition, or international diagnosis guidelines (16). Demographics and clinical data were obtained from medical records.

### Instruments

All patients included were assessed with the BI. This scale contains 10 items with varying weights measuring basic ADL. Two items regarding bathing and grooming are scored on a 2-point scale (0 or 1 points); six items regarding feeding, dressing, controlling one’s bladder, controlling one’s bowel, toilet use (getting onto and off the toilet), and stair climbing (ascending and descending stairs) are scored on a 3-point scale (0, 1, or 2 points). Two items regarding transferring (moving from a wheelchair to the bed and *vice versa*) and mobility (walking ability on a level surface) are scored on a 4-point scale (0, 1, 2, or 3 points). The BI score is the cumulative score of all 10 items, with a maximum score of 20 corresponding to complete independence, and a minimum score of 0 corresponding to total dependence. The Chinese version of the BI has been widely used in older populations (17). The BI has shown high internal consistency and inter-rater reliability, good concurrent validity, and adequate responsiveness among samples from various populations, such as stroke patients (18, 19) and patients receiving neurorehabilitation (20, 21). However, its psychometric properties in patients with dementia have not been examined yet.

Dementia severity was assessed using the CDR, where scores of 0 indicate no severity, 0.5 indicates questionable severity, 1 indicates mild dementia, 2 indicates moderate dementia, and 3 indicates severe dementia (22). The CDR is a widely used clinical scale for globally staging the level of dementia severity and is generally robust against the influence of cultural bias (23). It has established test–retest reliability, inter-rater reliability, concurrent validity, and can be administered by any trained personnel (24–26).

The Mini-Mental State Examination (MMSE) was used to measure cognitive function. It has a maximum score of 30, with lower scores indicating more severe cognitive impairment (27). The MMSE has established internal consistency (Cronbach’s *α* > 0.7), test–retest reliability (ICC 0.95), construct validity, and criterion validity (28, 29). Using the common cut-off value of <24, a recent systematic review summarized that sensitivity (0.75–0.88) and specificity (0.73–0.94) of the MMSE for detecting all-cause dementia in hospital samples were acceptable (30).

The Neuropsychiatric Inventory, Nursing Home version (NPI-NH) was used to assess the presence, frequency, and severity of behavioral and psychological symptoms of dementia (BPSD). The NPI-NH has established internal consistency (Cronbach’s *α* 0.64–0.80), test–retest reliability (ICC 0.89–0.93), inter-rater reliability, structural validity, and concurrent validity (Pearson correlation coefficient 0.54–0.67) (31, 32).

Because of the differences in the educational backgrounds of the healthcare professionals, their roles, and the staff mix among the study sites, the type of health care professionals who assessed the participants using the scales differed among the study sites. In mainland China and Taiwan, psychiatrists assessed the participants using the CDR and the MMSE, and graduate student nurses interviewed the staff members who took care of the participants to complete the NPI-NH and the BI. In South Korea, an experienced graduate student nurse interviewed the staff for the CDR and MMSE assessment, and registered nurses who were in charge of the participants completed the NPI-NH and the BI. In the Japanese hospital and long-term care facilities, registered nurses who were in charge of the participants filled out the CDR and MMSE sheets. In Japanese group homes, the staff members who lived with the participants filled out the CDR and MMSE sheets. In Thailand, a registered nurse and a clinical psychologist interviewed the staff for the CDR and MMSE assessment. The NPI-NH and the BI were assessed by the same method as for the CDR in Japan and in Thai. All the raters were trained in each region. The method regarding who performed the assessment using the above-mentioned instruments has been described previously (16).

To improve internal validity of the study, a standardized procedure of data collection was used. Skype conference was conducted to discuss all the issues that arose in the data collection phase. The quality of data collected has been monitored by the coinvestigators in each study site. Moreover, the principal investigator (the fifth author of this paper, Dr. Kiyoko Makimoto) flew to each study site at least once to make an on-site inspection during the data collection phase. Ten percent of the sample data had been checked for accuracy.

### Sample Size Calculations

For well-targeted tests, a sample size of 150 (n range, 108–243) was indicated to provide 99% confidence of item calibration ±0.5 logits. Conversely, for tests that are not well-targeted, a larger sample size (n = 243) is required for similar item location precision (33). Previous studies suggest that sample size greater than 300 may translate into an increasing possibility for type I errors, although both infit and outfit mean square statistics are relatively insensitive to sample size variation for polytomous data (34). Therefore, we used a random-sample of 300 out of the whole dataset to validate our results. We found that the results based on the random-sample were well consistent with those based on the whole dataset (see **Supplementary File Table S1**).

### Statistical Analysis

To ensure the requirement of a unidimensional construct for Rasch analysis, we first assessed the dimensionality of the scale using principal component analysis. If the proportion of variance explained by the Rasch dimension was >60% and the proportion of unexpected variance that accounted for the first contrast was <5%, the results were considered to support unidimensionality (33). The eigenvalue of the first contrast in the principal component analysis on standardized residuals less than 2.0 was considered ideal to support unidimensionality (33), and less than 3.0 was considered acceptable (35).

Internal consistency is indicated by person and item reliability which are analogous to Cronbach’s alpha in Rasch analysis. Person reliability < 0.8 implies that the instrument may not be sensitive enough to distinguish between high and low performers. Item reliability <0.9 implies that the sample is not large enough to confirm the item difficulty hierarchy of the instrument (33).

A nonsignificant log-likelihood chi-square value indicates good global fit to the Rasch model (33). A standard deviation (SD) of global fit residual >1.5 indicates poor model fit (36). Item fit was detected by the inlier-pattern-sensitive fit mean square (infit MnSq) and standardized Z (ZSTD). If the infit MnSq is acceptable (≤1.5), the ZSTD can be ignored (37). Misfitting items should be corrected first, before removing them from the scale (33). A large positive item residual correlation with other items (r > +.7) suggests highly local dependence (33).

A person–item map plotting item difficulty and person ability along a logit scale was used to demonstrate targeting of the BI to patients. A value of zero was allocated to the mean of item difficulty. The mean person location should approximate zero for a well-targeted tool (38). A well targeted instrument should contain items spanning across the full range of individual person locations. Thus, the map may help to identify gaps in coverage or redundant items. Gaps of more than 0.5 logits in the distribution of item difficulties indicate that items are needed to fill these gaps. If there are items with approximately the same difficulty, the items may be duplicative (33).

Threshold disordering was evaluated by visual inspection of category functions to determine whether the response probabilities are arranged in ascending order concordant with the categories. The logit distance between two adjacent categories should be <1.4 logits to show an appropriate threshold width (33). Narrow threshold width can usually be resolved by collapsing responses (15).

Differential item functioning (DIF) examines whether or not an item shows different endorsement probabilities across person groups with equal levels of capacity. In this study, DIF was identified by using log-odds estimators in Mantel for polytomies. Bonferroni’s correction was used for multiple comparisons. Because we observed obvious DIF across countries, we have then chosen Chinese and Japanese samples for further analysis of DIF by gender, age, and CDR, respectively. The sample size in the two sites was adequate for independent Rasch analysis. Regarding DIF across age, samples were divided into two groups (< 85-year-old and ≥85-year-old). Uniform DIF means that DIF is the same for all ability levels of the groups. Nonuniform DIF means that DIF differs with groups’ ability levels. Nonuniform DIF often requires removal of the item from the scale, whereas weighting or transitioning away from biased items is a potential solution for uniform DIF (39).

Although the underlying mechanisms of DIF are currently unclear, BPSD might contribute to the potential measurement bias when assessing basic ADL in patients with dementia. For example, distracting behaviors or refusal to eat may interfere with food intake (40); spatial agnosia may result in person with dementia being unable to locate the toilet, causing agitation and incontinence (41). To examine whether BPSD (measured by the NPI-NH in this study) has a negative impact on ADL abilities in the patients, Spearman’s rank correlation was furtherly conducted to identify negative correlations between NPI-NH and DIF item scores.

Rasch analysis was performed using WINSTEPS^®^ 4.0 (SWREG Inc., USA). Patient characteristics are presented as raw frequencies and percentages for categorical variables and as mean and SD for continuous variables. Spearman’s rank correlation was conducted with SPSS version 19.0 (IBM Corp, Armonk, NY, USA).

### Ethical Considerations

This research was approved by the ethics committee for each academic institution and care facilities (*e.g.*, in China, the study was approved by the Nanfang Hospital Ethical Committee), and written informed consent was obtained from all patients and/or their legal representatives if the patients did not have the capacity to consent. All necessary measures to safeguard participants’ anonymity and confidentiality of information were thoroughly followed.

## Results

In total, 644 eligible participants were included in the analysis (**Table 1**).

### Unidimensionality

The proportion of variances explained by the Rasch dimension was 72.9%. The proportion of unexpected variance accounted for by the first contrast (the largest secondary dimension) was 6.6%. The eigenvalues of the Rasch dimension and the first contrast were 27.0 and 2.4, respectively (see **Table 2**). Unidimensionality is a critical property of good measurement and a prerequisite to the summation of items within an instrument. These results indicated that the unidimensionality of the BI was not robust.

**Table 2:** Unidimensionality and Reliability after modification of the items in the Barthel Index (n = 644).

Variables	Original scale	Collapsing “mobility”	Removing “mobility”	Removing “stairs”	Combining “incontinence”	RC	RCC
Item Reliability	1.00	1.00	1.00	1.00	1.00	1.00	1.00
Person Reliability	0.88	0.88	0.88	0.88	0.88	0.88	0.87
Variance explained by Rasch dimension	72.9%	72.7%	74.7%	77.4%	74.1%	74.6%	75.6%
Variance explained by first contrast	**6.6%**	**6.1%**	**5.2%**	5.0%	**6.4%**	5.0%	4.7%
Eigenvalue of Rasch dimension	27.0	26.64	26.52	27.39	25.71	26.37	24.79
Eigenvalue of first contrast	**2.4**	**2.21**	1.84	1.78	**2.21**	1.80	1.53

Collapsing “mobility”, collapsing the second and the third categories of the “mobility” item; Removing “stairs”, removing the “stairs climbing” item; Combining “incontinence”, combining “controlling bowel” and “controlling bladder” into one item; RC, removing the “stairs climbing” item and collapsing the “mobility” item; RCC, removing the “stairs climbing” item, collapsing the “mobility” item, and combining “controlling bowel” and “controlling bladder” into one item. Values on the verge of meeting the requirements are marked in bold red.

The potential multidimensionality of the BI was not resolved by collapsing the misfit item “mobility” (collapsing categories with narrow threshold) or removing the “mobility” item. Considering that climbing stairs might not be necessary or possible for patients with dementia residing in LTCF, we removed the “stairs climbing” item. After removing the “stairs climbing” item, the unidimentionality for the scale was established (**Table 2**). However, the “mobility” item was still misfitting to the Rasch model (**Table 3**). After removing the “stairs climbing” item, collapsing categories with narrow threshold in the “mobility” item, and combining the “controlling bowel” and “controlling bladder” into one item, the unidimentionality (**Table 2**) and item fit (**Table 3**) of the scale became acceptable.

**Table 3:** The item location and results of item fit statistics of the Barthel Index for assessing patients with dementia (n = 644).

Item	Original scale			Removing “mobility”		Removing “stairs”		RC		RCC	
	Location (logit)	Infit MnSq	ZSTD	Infit MnSq	ZSTD	Infit MnSq	ZSTD	Infit MnSq	ZSTD	Infit MnSq	ZSTD
Bathing	3.73	0.63	−5.0	0.62	−5.3	0.65	−5.0	0.67	−4.8	0.66	-4.9
Grooming	2.53	0.71	−4.5	0.75	−3.8	0.70	−4.6	0.77	−3.4	0.75	-3.7
Stairs	1.54	1.22	3.2	**1.54**	7.4	/	/	/	/	/	/
Dressing	0.27	0.64	−6.8	0.74	−4.6	0.65	−6.5	0.77	−4.2	0.76	-4.4
Bladder	0.01	0.82	−3.1	0.86	−2.4	0.79	−3.6	0.90	−1.7	1.03	0.5
Bowel	−0.25	0.85	−2.5	0.87	−2.1	0.80	−3.5	0.90	−1.7		
Toilet	−0.02	0.59	−8.0	0.67	−6.0	0.58	−8.1	0.69	−5.7	0.71	-5.2
Feeding	−1.86	1.18	3.1	1.13	2.2	1.17	2.9	1.15	2.4	1.14	2.4
Mobility	−2.43	**1.86**	9.9	**/**	/	**2.03**	0.99	1.30	4.5	1.20	3.1
Transfer	−3.51	1.14	2.4	**1.58**	8.9	1.18	1.4	**1.61**	9.3	1.47	7.4

Removing “mobility”, removing the “mobility” item; Removing “stairs”, removing the “stairs climbing” item; Combining “incontinence”, combining “controlling bowel” and “controlling bladder” into one item; RC, removing the “stairs climbing” item and collapsing the second and the third categories of the “mobility” item; RCC, removing the “stairs climbing” item, collapsing the second and the third categories of the “mobility” item, and combining “controlling bowel” and “controlling bladder” into one item; infit MnSq, the inlier-pattern-sensitive fit mean square; ZSTD, standardized Z score. Values on the verge of meeting the requirements are marked in bold red.

### Internal Consistency

The values of person and item reliability were 0.88 and 1.00, respectively (**Table 2**), implying that the internal consistency of the BI was acceptable in assessing patients with dementia.

### Global Fit, Item Fit, and Local Dependence

Mean standardized fit residuals (−0.1) and corresponding standard deviations (0.94) fell within an acceptable range. The log-likelihood chi-square value was nonsignificant (χ^2^ = 8116, *P* = 0.78). The findings indicate an acceptable global fit to the Rasch model.

The item “mobility” was misfitting to the Rasch model (infit MnSq > 1.5). The results of item fit analysis are shown in **Table 3**. No local dependence was detected (**Supplementary File Table S2**).

### Targeting

**Figure 1:**
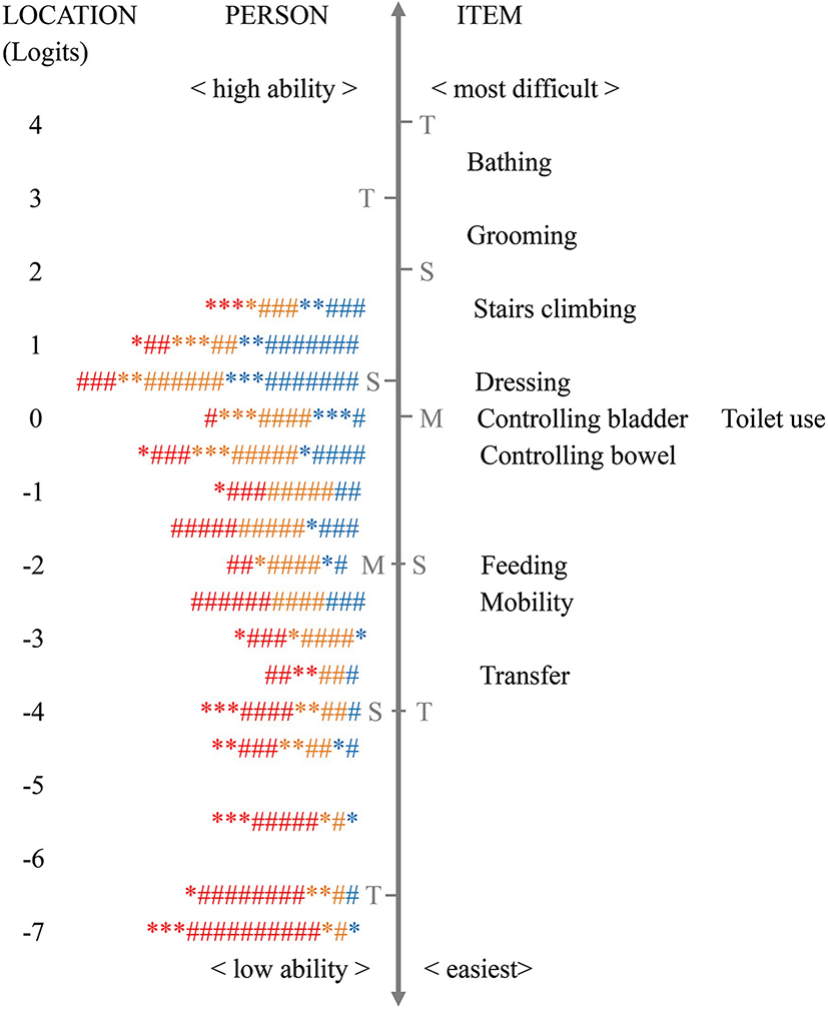
The Wright person–item map of the Barthel Index for assessing patients with mild (blue symbol), moderate (orange symbol), and severe dementia (red symbol) (n = 644). Note: each “*” = 1 person, and each “#” = 4 persons; M, mean person ability or mean item difficulty; S, one standard deviation; T, two standard deviations; CDR, Clinical Dementia Rating. The vertical line is a continuum representing the measures of person ability (left side) and item difficulty (right side), plotted in logit units. The person ability and item difficulty increase from the bottom to the top.

Targeting indicates whether the set of items is of appropriate difficulty for the target population. The mean person location was −2.27 (SD = 2.71) in the total samples and varied among patients with mild (mean = −0.82, SD = 2.43), moderate (mean = −1.68, SD = 2.22), and severe (mean = −3.23, SD = 2.39) dementia. The negative mean score of person location and the skewed person-item map (**Figure 1**) indicated that activities in the BI were difficult for patients to complete.

**Figure 1** shows that “bathing” was the most difficult activity, while “transfer” was the easiest (regarding item difficulty hierarchy, see also **Table 3**), and 28.4% of patients’ ability fell outside the coverage of the scale. On inspection of the person–item map, measurement gaps were observed between “controlling bowels” and “feeding” (> 1.0 logits), between “stair climbing” and “dressing”, and between “mobility” and “transfer” (> 0.5 logits, < 1.0 logits). The mean item difficulty of “controlling bladder” and “toilet use” overlapped at the same location, indicating potential redundancy of the items.

### Threshold Ordering

**Figure 2:**
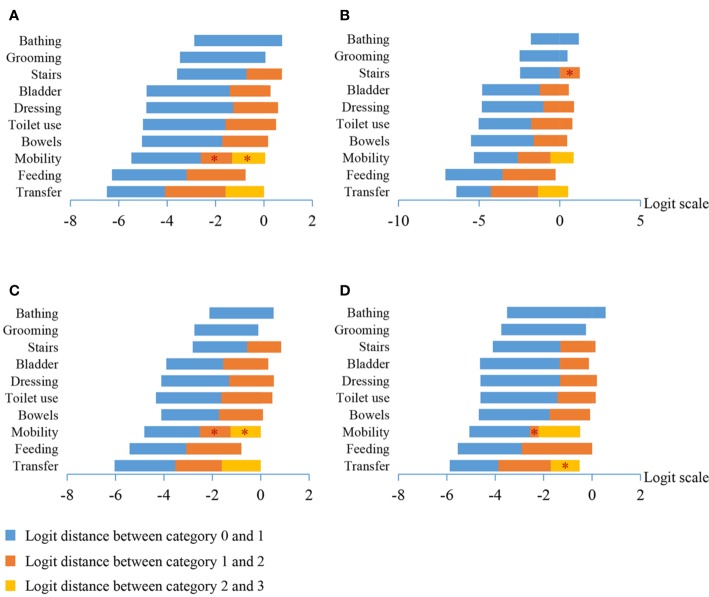
The logit distance between category thresholds for the 10 items of the Barthel Index in all the patients **(A)**, patients with mild dementia **(B)**, patients with moderate dementia **(C)**, and patients with severe dementia **(D)**. The red asterisk indicates a threshold width of less than 1.4 logits.

All BI items exhibited appropriately ordered thresholds, indicating that the respondents had no difficulty consistently discriminating between response categories. However, “mobility” for total samples (**Figure 2A**), “stair climbing” in patients with mild dementia (**Figure 2B**), “mobility” for patients with moderate to severe dementia (**Figures 2C, D**), and “transfer” for patients with severe dementia (**Figure 2D**) demonstrated narrow threshold widths (< 1.4 logits), indicating that collapsing the categories of the items or revision is needed.

### Differential Item Functioning

DIF indicates the potential measurement bias of an item. In the current study, when analyzing DIF related to countries, only three items (“grooming”, “dressing”, and “toilet use”) demonstrated invariant measurement traits, indicating that comparisons of BI scores between countries may not be reliable. Therefore, we further explored DIF by gender, age, and CDR in Chinese and Japanese samples, respectively. We have chosen Chinese and Japanese samples for further analysis, because the sample size in the two sites was adequate for independent Rasch analysis. DIF was found for “feeding”, “stairs climbing”, “mobility”, and “controlling bowel” in Japanese samples and for “feeding”, “stairs climbing”, “mobility”, and “transfer” in Chinese samples (**Table 4**). We removed the items with nonuniform DIF one by one and found that the unidimensionality and reliability were consistent with those of the original scale (**Supplementary File Table S1**).

**Table 4:** DIF for the items of the Barthel Index across gender, age, and CDR in Chinese and Japanese samples, respectively.

Item	M-China (n = 154)						Japan (n = 235)					
	Gender		Age		CDR		Gender		Age		CDR	
	UDIF (*df* = 1)	NUDIF (*df* = 3)	UDIF (*df* = 1)	NUDIF (*df =* 3)	UDIF (*df* = 2)	NUDIF (*df* = 5)	UDIF (*df* = 1)	NUDIF (*df* = 3)	UDIF (*df* = 1)	NUDIF (*df =* 3)	UDIF (*df* = 2)	NUDIF (*df* = 5)
Bathing	0.01	2.2	−0.4	4.2	0.02	2.5	1.5	1.6	−0.3	0.3	5.7	5.5
Grooming	0.3	3.2	0.2	1.3	2.5	3.2	0.7	1.1	0.2	0.4	0.03	0.4
Stairs	1.1	2.3	−1.1*	7.7	3.6	5.1	0.0	0.8	−0.9*	1.8	0.5	6.2
Toilet use	2.2	2.6	0.0	0.6	1.1	1.2	1.7	3.1	0.2	2.5	1.3	4.8
Dressing	2.6	4.6	0.2	1.9	3.4	4.3	0.5	12.3	0.2	10.8	5.0	14.1
Bladder	0.1	2.3	0.3	1.7	3.2	3.8	0.2	3.2	0.4	2.3	0.04	12.8
Bowels	0.6	3.5	0.5	4.7	1.4	3.2	3.3	20.8*	0.8*	20.6*	0.2	18.3*
Feeding	0.01	18.0*	1.3*	27.1*	0.8	17.4	0.6	35.7*	0.9*	42.1*	0.8	38.9*
Mobility	3.6	4.2	−1.0*	8.5	2.1	5.0	6.1*	24.6*	−1.3*	37.1*	0.2	26.7*
Transfer	1.6	13.2*	−0.5	12.6*	1.5	12.8	0.1	10.6	−0.4	11.6	5.7	16.7

The values in the table are Mantel–Haenszel or Mantel χ^2^. *P < 0.05 (df = 1), P < 0.02 (df = 2), P < 0.008 (df = 3), and P < 0.004 (df = 5), after Bonferroni correction of P value. DIF, Differential Item Functioning; CDR, clinical dementia rating; M-China, mainland China; UDIF, Uniform DIF; NUDIF, Nonuniform DIF.

Regarding DIF items, in the Japanese samples, scores for “feeding” were negatively related to scores for “disinhibition” (r = −0.46, P < 0.01), and scores for “controlling bowel” were negatively related to scores for “disinhibition” (r = −0.44, P < 0.01), “agitation” (r = −0.32, P < 0.05), and “aggression” (r = −0.27, P < 0.01). In the Chinese samples, no negative correlations between NPI-NH and DIF item scores were observed.

Based on results of Rasch analysis, we provided suggestions for future refining of the instrument when assessing patients with dementia, including filling measurement gaps (*e.g.*, the gap between the “feeding” and “controlling bowel” items), modifying redundant items (*e.g.*, “controlling bladder” and “toilet use”), collapsing narrow thresholds for the “mobility”, “transfer”, and “stair climbing” items, and considering potential interactions between BPSD and ADL abilities (**Supplementary File Table S3**). Although modifying the instrument was not the purpose of the current study, we tried to do a limited revision of the scale based on these suggestions. For example, after removing the “stairs climbing” item, collapsing categories with narrow threshold width in the “mobility” item, and combining the “controlling bowel” and “controlling bladder” into one item, unidimentionality and item fit of the revised scale were better than those of the original scale (see **Table 2** and **Table 3**).

## Discussion

Assessment of ADL in patients with dementia is important for evaluating interventions and determining care needs. To the best of our knowledge, the BI, a widely used basic ADL scale, has not been previously examined for assessing patients with dementia. One strength of the current study is that, by using Rasch analysis, a rigorous technique focusing on individual items, we evaluated the application of the BI for assessing the patients and provided suggestions for future refining of the instrument in assessing basic ADL in patients with dementia. Another strength is that our data were obtained from a multi-country study on the prevalence of BPSDs, enabling examination of DIF across countries, and exploration of the potential contributions of interactions between BPSD and ADL to item bias.

Unidimensionality is a prerequisite to the summation of item scores within an instrument (33). There is a discrepancy relating to the unidimensional qualities of the BI in previous studies (42–44). By using Rasch analysis, researchers report that the BI is not unidimensional in stroke patients (45), patients receiving neurologic rehabilitation (13), and patients in acute care settings (46). “Controlling bowel” and “controlling bladder” misfit the Rasch model based on the BI scores from neurologic rehabilitation population (13), while more than half of the items in the BI misfit to the Rasch model for older acute medical patients (46). Our findings suggest that the robustness of the unidimensionality of the BI should be established for assessing patients with dementia.

In this study, the “mobility” item exhibited misfitting to the current Rasch model, and the “mobility” and “stairs climbing” showed narrow threshold width. These problems might have compromised unidimensionality of the BI. Previous studies based on Rasch analysis also suggest that the adjacent rating categories “wheel chair independent” and “walks with help” in the “mobility” item might be ambiguous since many patients use a wheelchair while able to walk short distances (45). However, when we removed the misfit item “mobility”, the “stair climbing” item and the “transfer” item became misfit to the Rasch model. When we removed the “stairs climbing” item and collapsed categories with narrow threshold width of the “mobility” item, unidimentionality and item fit of the scale were established. These findings suggest that the mobility ability might not be helpful for constructing basic ADL capability in patients with dementia. This may have occurred because the limitations of basic ADL among patients are most often the result of cognitive decline, while physical abilities of the patients remain relatively intact until the late stages of the disease (47). The discordance between mobility ability and basic ADL capability compromises the utility of the scale in the patients, particularly when there are interactions between BPSD (*e.g.*, nighttime behavior or wandering behavior) and mobility ability. Since 2017, BI summary scores have been used as an appraisal of eligible beneficiaries of public long-term care insurance in China (48). However, maintenance of misfit items, such as “mobility”, may lead to distorted results used for assessing ADL dependence in applicants with dementia. This finding has implications for long-term care insurance policy, driven by reported levels of basic ADL capacity.

Our findings demonstrated that the BI was not well targeted to patients with item difficulty exceeding person abilities, and patients with more severe dementia demonstrated greater person–item mismatch. Moreover, patients with mild to severe severity of dementia coexisted in the same locations, suggesting that basic ADL might not be specific for differentiating severity of dementia. The item difficulty hierarchy of the BI in the present study might help in understanding the order of loss of ADL abilities in the patients and provide useful information for observing and identifying potential functional impairment among the patients. For example, inability to perform the easiest ADLs (such as transfer) indicates severe functional dependence of the patients, whereas inability to perform only the most difficult ADLs (such as bathing) suggests mild functional dependence of the patients. The items with higher difficulty level tend to be lost at the earlier stage than those with lower difficulty level. Previous studies suggest that the order of loss of ADLs for nursing home residents is: bathing, dressing, transfer and locomotion, toileting, and finally eating (49). Our findings suggest that patient with dementia also tend to be dependent for bathing, while transfer and mobility might be the last ability lost in the patient.

Identifying DIF is a critical step for improving measurement. DIF for the BI has not been previously reported (46). In the current study, violation of measurement invariance across countries suggests that BI scores should not be compared between cultures. Although the underlying mechanisms of DIF in patients are currently unclear, BPSD, cultural factors (*e.g.*, caregivers are expected to provide assistance as an expression of caring in Chinese culture) (50), multiple medications (*e.g.*, prevalence of psychotropic prescriptions) (51), and interactions between patients and their caregivers (52) may contribute to DIF. Available studies have indicated an association between presence/degree of behavioral disturbance (*e.g.*, apathy, paranoia, agitation) and physical ADL performance (53–55). The correlations between scores of the BI and the NPI-NH found in the present study suggest that neuropsychiatric symptoms should be taken into consideration when assessing basic ADL abilities of patients with dementia.

Several limitations of the present study should be noted. First, since the BI and the NPI-NH are informant-based assessments, scoring could be affected by information provided by the informant and memory bias. Second, correlations between specific NPI-NH and BI items might be unstable in different samples due to different prevalence rates of BPSDs in the samples and memory bias. Observation of patients’ ADL ability and BPSD by trained research assistants may help to overcome this limitation in future studies. Third, the MMSE is a norm-based psychometric measure which compares individual functioning to a reference group, therefore, confounding factors, such as education level and floor effect, might impact this norm-based measures (30). Adjusting for confounding should be taken to improve diagnostic accuracy in our future research, although 75.6% of the subjects were at moderate to severe stage of dementia in this study. Furthermore, previous studies suggest that CDR 0.5 may describe a broader population that includes subjects with mild cognitive impairment and mild dementia (56). We therefore excluded subjects with CDR score of 0.5 from this study. Improved staging tools should be used to accurately differentiate early stage of dementia from mild cognitive impairment in future studies.

## Conclusions and Implications

Using Rasch analysis, the current study revealed that the utility of the BI for assessing basic ADL in patients with dementia is compromised by person–item mistargeting, measurement gaps, redundant items, narrow threshold width, and item bias. Thus, the BI summary score may not help in differentiating ADL levels among patients. Mobility may not be helpful in determining ADL capability of the patients. Comparisons of BI scores between cultures may not be reliable due to item bias across countries. We will not suggest using the instrument in patients with dementia without future refining to improve its performance for the patients. Dementia specific instruments for ADL measurement, such as the ALD-IS (57), might help to capture transition across the spectrum of basic ADL abilities of patients with dementia. Therefore, we provided useful information to help guide future revision or development of the ADL instrument for assessing basic ADL in patients with advanced dementia based on findings from the current Rasch analysis.

## Data Availability Statement

The datasets generated for this study are available on request to the corresponding author.

## Ethics Statement

This research was approved by the ethics committee for each academic institution and care facilities (e.g., in China, the study was approved by the Nanfang Hospital Ethical Committee), and written informed consent was obtained from all patients and/or their legal representatives if the patients did not have capacity to consent.

## Author Contributions

Study concept and design: XL and KM. Acquisition of data: LD, JW, YY, and HW. Analysis and interpretation of data: YY, LD, and XL. Drafting of the manuscript: XL, LD, and YY. All authors contributed to revising the manuscript critically for important intellectual content, and final approval of the version to be submitted. YY and LD contributed equally to the work.

## Funding

XL has received support from the Science and Technology Planning Project of Guangdong Province, China (Grant Number 2017A020215041) and the National Natural Science Foundation of China (Grant Number 81400868). Dr. Makimoto has received funding from the JSPS KAKENHI (Grant Number JP26305018).

## Conflict of Interest

The authors declare that the research was conducted in the absence of any commercial or financial relationships that could be construed as a potential conflict of interest.
