# Supplementary material for: Is Barthel Index Suitable for Assessing Activities of Daily Living in Patients With Dementia?
Source: Front Psychiatry. 2020 May 8;11:282. doi: 10.3389/fpsyt.2020.00282 (PMC7225343; doi:10.3389/fpsyt.2020.00282)
Supplement: Supplementary file 1 [file Table_1.docx]

**Supplementary Table S1.** Unidimensionality and Reliability in random samples and after removing DIF items in Japan and China samples.

| Variables | Random samples  (n=300) | Japan samples (n=235) | | | |  | M-China samples (n=154) | | |
| --- | --- | --- | --- | --- | --- | --- | --- | --- | --- |
|  |  | Original | Removing Mobility | Removing Feeding | Removing Bowels |  | Original | Removing  Feeding | Removing Transfer |
| Person Reliability | 0.88 | 0.88 | 0.87 | 0.88 | 0.87 |  | 0.88 | 0.88 | 0.84 |
| Item Reliability | 1.00 | 1.00 | 1.00 | 1.00 | 1.00 |  | 0.99 | 0.99 | 0.99 |
| Variance explained by Rasch dimension | 72.7% | 73.4% | 74.9% | 75.2% | 75.1% |  | 75.3% | 79.0% | 73.0% |
| Variance explained by first contrast | 6.8% | 6.1% | 5.3% | 6.7% | 6.3% |  | 5.9% | 5.8% | 6.2% |
| Eigenvalue of Rasch dimension | 26.66 | 27.58 | 26.87 | 27.22 | 27.20 |  | 30.46 | 33.77 | 24.38 |
| Eigenvalue of first contrast | 2.50 | 2.52 | 1.90 | 2.45 | 2.27 |  | 2.39 | 2.49 | 2.07 |

Note: DIF=differential item functioning. M-China=mainland China.
