# Supplementary material for: Is Barthel Index Suitable for Assessing Activities of Daily Living in Patients With Dementia?
Source: Front Psychiatry. 2020 May 8;11:282. doi: 10.3389/fpsyt.2020.00282 (PMC7225343; doi:10.3389/fpsyt.2020.00282)
Supplement: Supplementary file 2 [file Table_2.docx]

**Supplementary Table 2**. Residual correlation matrix of the items of Barthel Index for assessing local dependence (n=644).

| Item | Bowels | Bladder | Grooming | Toilet use | Feeding | Transfer | Mobility | Dressing | Stairs | Bathing |
| --- | --- | --- | --- | --- | --- | --- | --- | --- | --- | --- |
| Bowels | \ | 0.429 | -0.043 | 0.057 | 0.026 | -0.290 | -0.436 | -0.060 | -0.361 | -0.036 |
| Bladder | 0.429 | \ | -0.035 | 0.086 | -0.089 | -0.286 | -0.371 | -0.095 | -0.307 | -0.058 |
| Grooming | -0.043 | -0.035 | \ | 0.046 | 0.077 | -0.075 | -0.295 | -0.030 | -0.220 | 0.096 |
| Toilet use | 0.057 | 0.086 | 0.046 | \ | -0.134 | -0.149 | -0.177 | -0.128 | -0.216 | 0.026 |
| Feeding | 0.026 | -0.089 | 0.077 | -0.134 | \ | -0.255 | -0.416 | -0.029 | -0.242 | -0.079 |
| Transfer | -0.290 | -0.286 | -0.075 | -0.149 | -0.255 | \ | 0.083 | -0.149 | 0.022 | -0.104 |
| Mobility | -0.436 | -0.372 | -0.296 | -0.177 | -0.416 | 0.083 | \ | -0.121 | 0.3672 | -0.164 |
| Dressing | -0.060 | -0.095 | -0.030 | -0.127 | -0.029 | -0.149 | -0.121 | \ | -0.129 | -0.007 |
| Stairs | -0.361 | -0.307 | -0.220 | -0.216 | -0.242 | 0.002 | 0.367 | -0.129 | \ | -0.020 |
| Bathing | -0.036 | -0.058 | 0.096 | 0.026 | -0.079 | -0.104 | -0.164 | -0.007 | -0.020 | \ |
