# Supplementary material for: Is Barthel Index Suitable for Assessing Activities of Daily Living in Patients With Dementia?
Source: Front Psychiatry. 2020 May 8;11:282. doi: 10.3389/fpsyt.2020.00282 (PMC7225343; doi:10.3389/fpsyt.2020.00282)
Supplement: Supplementary file 3 [file Table_3.docx]

**Supplementary Table S3**. Suggestions for modifying the items of the Barthel index in assessing patients with dementia.

| **Items** | **Categories** | **Suggestions** |
| --- | --- | --- |
| Bathing | 0=dependent | (1) Add easer categories to improve targeting to patients with dementia, such as |
|  | 1=independent (or in shower) | “washes part of body only, but cannot bathe rest of body”. |
|  |  | (2) Consider impact of cognitive impairment or BPSD on bathing ability, such as “needs supervision or reminders to bath completely”, or “cannot wash self but is cooperative with those who bathe her or him”. |
| Grooming | 0=needs help with personal care | (1) Add more categories to improve targeting to patients with dementia, such as |
|  | 1=independent face/hair/teeth/shaving | “needs physical help, but can remain well-groomed after help from others”, |
|  | (implements provided) | “keeps face and hands clean without physical help”, “brushes hair without physical help”, or “cleans and cut fingernails without physical help”. |
| Stair | 0=unable | (1) Consider removing the item ‘stair climbing’ from the scale, because of climbing |
| climbing | 1=needs help (verbal, physical, carrying aid) | stair may not applicable in long-term care facilities. |
|  | 2=independent up and down |  |
| Dressing | 0=dependent | (1) Add more categories to improve measuring gaps in assessing patients with |
|  | 1=needs help, but can do about half unaided | dementia, such as “dresses without help if clothes need no fastening or buttoning”. |
|  | 2=independent (including buttons, zips, laces, etc.) | (2) Consider impact of cognitive impairment or BPSD on bathing ability, such as “dresses completely with supervision, but without physical help”. |
| Controlling | 0=incontinent (or needs to be given enemata) | (1) Combine bowel and bladder into one item to modify overlapping of item difficulty |
| bowel | 1=occasional accident (once/week) | *Example from Katz index of independent in activities of daily living:* |
|  | 2=continent | 0=partially or totally incontinence of bowel or bladder. |
| Controlling | 0=incontinent, or catheterized and unable to | 1=exercise completely control over urination and defecation. |
| bladder | manage | (2) modify categories of toilet use to target to patients with dementia, such as: |
|  | 1=occasional accident (max. once per 24 hours) | “needs supervision or to be reminded, but no physical help”, “needs to be taken to the |
|  | 2=continent (for over 7 days) | toilet and given assistance”, or “needs to be taken to the toilet and given assistance” |
| Toilet use | 0=dependent | (3) Or further combine incontinent and toilet use into one item. |
|  | 1=needs some help, but can do something alone | *Example from ADCS-ADL:* |
|  | 2=independent (on and off, dressing, wiping) | 0=needed physical help, and was usually incontinent |
|  |  | 1=needed physical help, and was usually continent |
|  |  | 2=needed supervision, but no physical help |
|  |  | 3=did everything necessary without supervision or help |
| Feeding | 0=unable | (1)Add easier categories to fill measuring gap and improve targeting, such as |
|  | 1=needs help cutting, spreading butter, etc. | “uses a fork or spoon, but not chopsticks or a knife” |
|  | 2=independent (food provided within reach) | (2) Consider impact of interactions between BPSD and feeding ability, such as ‘if her or him eaten appropriately using correct cutlery”, or “used fingers to eat” |
| Mobility | 0=immobile | (1) Collapsing narrow categories in the item ‘mobility’, such as collapsing “wheelchair |
|  | 1=wheelchair independent, including corners, etc. | independent, including corners” and “walks with help of one person” into “ambulates |
|  | 2=walks with help of one person (verbal or physical) | with assistance of another person or railing or walker or wheel chair”. |
|  | 3=independent (but may use any aid, e.g., stick) | (2) Or combine the items into one. *Example from ADCS-ADL:* |
| Transfer | 0=unable – no sitting balance | 0=required physical help to walk or transfer |
|  | 1=major help (one or two people, physical), can sit | 1=transferred from bed to chair without help |
|  | 2=minor help (verbal or physical) | 2=mobile across a room without physical help |
|  | 3=independent | 3=mobile outside of home without physical help |

**Note**: ADCS-ADL*=* Alzheimer's disease cooperative study - activities of daily living. BPSD=behavioral and psychological symptoms of dementia.
